# Synchronous or metachronous breast and colorectal cancers in younger-than-average-age patients: a case series

**DOI:** 10.1093/oncolo/oyae114

**Published:** 2024-06-10

**Authors:** Jordyn Silverstein, Francis Wright, Dalila Stanfield, Amy Jo Chien, Jasmine M Wong, John W Park, Amie Blanco, Katherine Van Loon, Chloe E Atreya

**Affiliations:** Division of Hematology/Oncology, Department of Medicine, University of California, San Francisco (UCSF), San Francisco, CA 94143, United States; School of Medicine, University of California, San Francisco, San Francisco, CA 94143, United States; UCSF Helen Diller Family Comprehensive Cancer Center, San Francisco, CA 94143, United States; Division of Hematology/Oncology, Department of Medicine, University of California, San Francisco (UCSF), San Francisco, CA 94143, United States; UCSF Helen Diller Family Comprehensive Cancer Center, San Francisco, CA 94143, United States; Department of Surgery, UCSF, San Francisco, CA 94143, United States; Division of Hematology/Oncology, Department of Medicine, University of California, San Francisco (UCSF), San Francisco, CA 94143, United States; UCSF Helen Diller Family Comprehensive Cancer Center, San Francisco, CA 94143, United States; UCSF Helen Diller Family Comprehensive Cancer Center, San Francisco, CA 94143, United States; Cancer Genetics and Prevention Program, UCSF, San Francisco, CA 94143, United States; Division of Hematology/Oncology, Department of Medicine, University of California, San Francisco (UCSF), San Francisco, CA 94143, United States; UCSF Helen Diller Family Comprehensive Cancer Center, San Francisco, CA 94143, United States; Division of Hematology/Oncology, Department of Medicine, University of California, San Francisco (UCSF), San Francisco, CA 94143, United States; UCSF Helen Diller Family Comprehensive Cancer Center, San Francisco, CA 94143, United States

**Keywords:** young, colorectal, breast cancer, genetic

## Abstract

**Background:**

The incidence of breast and colorectal cancer (CRC) in younger-than-average-age patients is rising and poorly understood. This is the largest study on patients with both cancers who are less than 60 years old and aims to characterize demographic, clinicopathologic, and genetic features and describe therapeutic dilemmas and management strategies.

**Materials and Methods:**

This is a retrospective medical records review of patients at the University of California San Francisco with both primary breast and CRC before age 60.

**Results:**

Fifty-one patients were identified; 41 had detailed medical records. Median age of diagnosis with breast cancer was 43 (range 27-59) and CRC was 50 (28-59). Most were Caucasian (38, 74.5%) and never smokers (23, 56.1%); about half were current alcohol consumers (20, 48.8%) and about one-third had sedentary jobs (14, 34.1%). Average BMI was 25.8 (range: 14-49), and 30% were overweight or obese. Breast was the first cancer diagnosed in 36 patients (70.6%) and 44 (86.3%) had a metachronous CRC diagnosis. Breast cancer was early stage (0-2) in 32 (78.0%) patients whereas CRC was split between early stage (1-2) in 14 (34.1%) and later stage (3-4) in 19 (46.2%). Ten patients (24.3%) had a known germline mutation, although 23 (56.1%) had a family history of cancer in a first-degree relative.

**Conclusion:**

Younger patients with both breast and CRC are a unique cohort, often without known risk factors. Alcohol consumption and sedentary jobs were the most common risk factors, and about one-quarter had a known genetic predisposition. Comanagement of both cancers requires individualized, multidisciplinary care.

## Teaser

Little is known about the proportion of young patients affected by dual diagnoses of breast cancer and colorectal cancer. This is the largest study to date on patients with both cancers who are younger than 60 years.

**Implications for Practice**

This is the largest case series on younger patients with both breast and colon cancer. It characterizes the demographics, risk factors, and genetic features to help understand this cohort. Additionally, the study describes management nuances of treating both cancers that could help guide the treatment of patients in the future faced with this dual diagnosis.

## Introduction

Breast cancer and colorectal cancer (CRC) are 2 of the 3 most common cancers. In the United States, these cancers account for approximately 30% and 8% of new cancer cases in women each year, respectively.^1^ The median age at diagnosis is 63 years old for breast cancer and 67 years old for CRC.^2,3^ However, approximately 32% of breast cancer cases and 10% of CRC cases are considered young-onset, with a diagnosis before the age of 50.^4,5^ Among early-onset cancers, breast has the highest number of incident cases and gastrointestinal cancers have the fastest-growing incidence rates.^4-8^ Overlapping risk factors for both breast cancer and CRC include physical inactivity, central obesity, and alcohol consumption.^7^ Limited information is known about what proportion of young patients are impacted with dual diagnoses of breast cancer and CRC.

The frequency of multiple primaries in a cancer population is estimated to be around 2%-17%.^9^ The largest study of patients with both breast cancer and CRC used Surveillance, Epidemiology, and End Results (SEER) data from 1988 to 2007 to describe outcomes for 4835 patients; among this group, 75% were diagnosed with either cancer after age 65.^10^ This study did not investigate genetic risk factors, family history, behavioral risk factors, or treatment-related factors. To date, the literature is mostly comprised of case series (105 and 299 patients) and case reports of colon and breast cancer in individual patients, all of which have an average age around 65.^11-18^ Only a few case reports exist for young patients and only 5 case reports on patients less than 60 years old were identified.^15,19^ Additionally, only one case demonstrated a genetic mutation (CHEK2*1100delC) to explain both a hereditary breast and CRC phenotype.^20^

In light of the absence of data on patients less than 60 years old with a dual diagnosis of breast cancer and CRC, our aims were to describe: (1) demographic and known cancer risk factors, (2) clinicopathologic features and somatic and germline mutational profiles, and (3) clinical management and outcomes of younger-than-average patients with both breast cancer and CRC. We hypothesized that systematic characterization of these patients would inform understanding of the unique diagnostic and nuanced therapeutic implications for this population.

## Materials and methods

### Study design and study population

This study is a retrospective medical records review of patients at the UCSF Helen Diller Family Comprehensive Cancer Center. The study was approved by the University of California, San Francisco (UCSF) Human Research Protection Program (number 22-36184).

Following approval, patients were identified by physician recall as well as computational data extraction to find patients within UCSF’s Cancer Registry as well as the Cancer Genetics Registry who were diagnosed with histologically confirmed colorectal adenocarcinoma and any breast lesions that would require treatment (including ductal carcinoma in situ [DCIS] and growing lesions) before age 60 at UCSF during 2001-2022. The age threshold of 60 was chosen to maximize the number of patients included in this study with the rationale that it is below the average age of diagnosis of either cancer and, using this age cutoff, the first cancer was diagnosed at age <50 in the majority of patients. Additionally, prior research on this population included only rare patients who are under 60 years old, so younger-than-average patients are understudied.

### Data collection

Data were abstracted from patient charts by a single reviewer (J.S.) and discussed with a board-certified oncologist (C.E.A.). The data cutoff was December 19, 2022. The variables that were abstracted included age, self-reported gender identity, race/ethnicity, birthplace, smoking and alcohol history, occupation, body mass index (BMI) at diagnosis of first cancer or earliest BMI in the medical record, past medical history, order of cancer diagnosis and whether metachronous or synchronous, date/age of diagnosis, cancer stage at diagnosis, hormone receptor status at diagnosis, histology, grade, tumor location, known germline mutation, family history in a first-degree relative, MMR status, MSI status, BRCA status, and date of last contact or death, and whether they are deceased or alive. During chart review, additional treatment details were collected for patients where there was clinical decision-making related to a patient receiving treatment for both primary tumors simultaneously. The variables list was approved by both a CRC oncologist (C.A.) as well as breast oncologists (A.J.C., J.C., J.W.) for completeness. Patients with synchronous cancers were defined as diagnosed of both primary tumors within 6 months of each other. Patients with metachronous cancers were defined as having a diagnosis of each cancer more than 6 months apart.

### Statistical variables and analysis

Descriptive statistics were used to summarize demographic, clinical, pathologic, and genetic data, as well as the prevalence of various lifestyle factors. All data analyses were performed using Excel and STATA.

## Results

### Demographic information

A total of 51 patients were identified with both breast and CRC diagnosed before the age of 60, and detailed information was available for 41 of these patients. Nine were identified by physician recall, 17 from the UCSF Cancer Registry, and 30 from the Cancer Genetics Registry; of the 41 patients, 5 overlapped between physician recall and the UCSF Cancer Registry. The majority of breast cancers were diagnosed before 2010 (*n* = 27, 65.8%) while the majority of CRC cases were diagnosed after 2010 (27, 65.8%).

Only one patient included in this cohort self-identified as a man (1.9%), and all other patients self-identified as a woman (Table 1). The median age of breast cancer diagnosis was 43 (range 27-59), and the median age of CRC diagnosis was 50 (range 28-59). Out of 51 total patients, 10 patients (19.6%) had both cancers diagnosed before age 50, and 41 (80.4%) had the first cancer diagnosed before age 50. Only 10 patients (19.6%) had both cancers diagnosed between age 50 and 60. Most patients were Caucasian (38, 74.5%) and never smokers (23, 56.1%). Nearly half were current alcohol consumers (20, 48.8%). The average BMI was 25.8 (range: 14-49), and 46% (19) were overweight or obese at diagnosis of the first cancer or the earliest BMI recorded after the first cancer. Ten patients (24.3%) were known to be born outside of the United States, originating from Pakistan (*n* = 2), Japan, the Philippines, France, Israel, Ghana, Vietnam, Burma, and Mexico.

**Table 1.** Baseline demographic and clinical information.

Younger-onset breast and CRC (*n* = 51)	
Gender (%)	
Men	1 (2.0)
Women	50 (98.0)
Age at breast cancer diagnosis	
Median, years (IQR)	43 (39-49)
Missing	1 (2.0)
Age at CRC diagnosis	
Median, years (IQR)	50 (44-55)
Race/ethnicity (%)	
Caucasian/non-Hispanic	38 (74.5)
Caucasian/Hispanic	2 (3.9)
Asian	9 (17.6)
Other^a^	1 (2.0)
Missing	1 (2.0)
Cancer first diagnosed	
Breast	36 (70.6)
Colorectal	13 (25.5)
Missing	2 (3.9)
Metachronous or synchronous	
Metachronous	44 (86.3)
Synchronous	7 (13.7)
Younger-onset breast and CRC with detailed medical records (*n* = 41^b^)	
BMI	
Median (IQR)	25 (22-28)
Missing (%)	6 (14.6)
Birthplace (%)	
United States	10 (24.3)
Outside United States	10 (24.3)
Missing	21 (51.2)
Alcohol use (%)	
Current consumer	20 (48.8)
Prior consumer	4 (9.8)
Never consumer	14 (34.1)
Missing	3 (7.3)
Tobacco use (%)	
Current smoker	4 (9.8)
Prior smoker	11 (26.8)
Never smoker	23 (56.1)
Missing	3 (7.3)
Breast cancer	
Stage at diagnosis	
0	11 (26.7)
1	12 (30.3)
2	9 (21.9)
Missing	9 (21.9)
Histology	
DCIS	13 (31.7)
LCIS	1 (2.4)
IDC	17 (41.5)
ILC	1 (2.4)
Fibroepithelial lesion^c^	1 (2.4)
Missing	8 (19.5)
Location of tumor	
Left breast	17 (41.5)
Right breast	18 (43.9)
Both breasts	1 (2.4)
Missing	5 (12.2)
CRC	
Stage at diagnosis	
1	8 (19.5)
2	6 (14.6)
3	9 (21.9)
4	10 (24.4)
Missing	8 (19.5)
Grade^d^	
Poorly differentiated	4 (9.8)
Moderately differentiated	19 (46.3)
Well differentiated	6 (14.6)
Missing	12 (31.3)
Location of tumor	
Right colon (ascending, cecum)	9 (21.9)
Transverse colon	5 (12.2)
L colon (descending, sigmoid)^e^	7 (17.1)
Colon NOS	6 (14.6)
Rectum	13 (31.7)
Missing	1 (2.4)

^a^Other race/ethnicity was Lebanese.

^b^Forty-one out of the 51 patients had detailed information in the medical record.

^c^Fibroepithelial lesion was included since it was an actively growing lesion that impacted decision-making.

^d^Well to moderately differentiated was considered well differentiated, poorly to moderately differentiated was considered poorly differentiated.

^e^If a patient had a rectosigmoid tumor that was treated with a hemicolectomy then it was considered a sigmoid rather than a rectal primary.

Abbreviations: IQR, inter-quartile range; BMI, body mass index; DCIS, ductal carcinoma in situ; LCIS, lobular carcinoma in situ; IDC, invasive ductal carcinoma; ILC, invasive lobular carcinoma; NOS, not otherwise specified.

### Breast cancer clinicopathologic information

Breast cancer was the first cancer diagnosed in 36 patients (70.6%), and 44 out of the 51 (86.3%) had a metachronous diagnosis with CRC. There were 7 (13.7%) cases of synchronous breast and CRC. The breast cancers were predominantly early stage 0 (11, 26.7%), stage 1 (12, 30.3%), and 2 (9, 21.9%). The most common breast cancer types were invasive ductal carcinoma (17, 41.5%) and DCIS (13, 31.7%), and they were split between the right (43.9%, 18) and left breast (41.5%, 17). One patient had cancer in both breasts and 5 (12.2%) were missing location.

### Colorectal cancer clinicopathologic information

The CRC cases were evenly distributed across all stages at the time of diagnosis, stage 1 (8, 19.5%), stage 2 (6, 14.6%), stage 3 (9, 21.9%), and stage 4 (10, 24.4%). All CRC cases were adenocarcinoma (34 confirmed patients, 7 with missing data from the chart) and mostly moderately differentiated (19, 46.3%). The tumors occurred in the right colon (9, 21.9%), transverse colon (5, 12.2%), left colon (7, 17.1%), and rectum (13, 31.7%). Only one patient had a known history of inflammatory bowel disease. Fourteen patients (34.1%) had sedentary jobs including advertising, consulting, designers, and office workers; and 2 (4.9%) were restaurant workers.

### Genetic information

Twenty-three patients (56.1%) had a family history of cancer in a first-degree relative, and 16 (39.0%) had a first-degree family member with breast, CRC, or both (Table 2). One patient was adopted with an unknown family history and 13 (31.7%) had documentation of no family history in a first-degree relative. Ten patients (24.3%) had a known germline mutation, and all of these were patients who had a known family history of cancer in a first-degree relative (8) or a missing family history (2). Germline mutation data were missing from the charts of 12 patients (29.3%). The known germline mutations were Lynch syndrome (5, 12.2%) and BRCA mutation (5, 12.2%). Of the patients with Lynch syndrome, there were mutations in MSH2 (2), MSH6 (2), and PSM2 (1). Two patients had BRCA1 and 3 had BRCA2 mutations. Nineteen patients (46.3%) had no identifiable germline mutations after testing.

**Table 2.** Genetic risk factors.

Younger-onset breast cancer and CRC (*n* = 41)	
Family history of cancer in a first-degree relative (%)	
Yes	23 (56.1)
No	13 (31.7)
Missing or unknown	5 (12.2)
Type of cancer in first-degree relative (*n* = 23)	
Breast	9 (39.1)
Colorectal	6 (26.0)
Both breast and CRC	1 (43.4)
Known germline mutation (%)	
Yes	10 (24.3)
No	19 (46.3)
Missing	12 (29.3)
Type of germline mutation (*n* = 10)	
Lynch	5 (50.0)
BRCA^b^	5 (50.0)

^a^Lynch Syndrome consisted of mutations in MSH2, MSH6, and PMS2.

^b^BRCA mutations: BRCA-1 (*n* = 2) and BRCA-2 (*n* = 3).

Seven patients (17.1%) had an additional cancer other than breast and CRC including endometrial cancer (4), lung cancer (1), follicular lymphoma (1), and uterine cancer (1). Six patients (14.6%) had no known family history of cancer in a first-degree relative and no known germline mutation.

### Therapeutic dilemmas and management strategies

**Figure 1.**
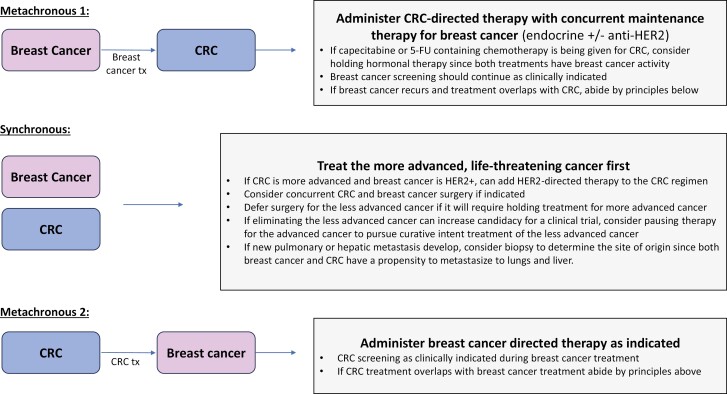
Workflow of clinical decision-making with breast and colorectal cancers diagnosed metachronously or synchronously. Abbreviations: CRC, colorectal cancer; tx, treatment.

A subgroup of patients (7 out of 41, 17.1%) had either synchronous diagnoses or simultaneous treatment of both cancers due to either a new cancer diagnosis or new recurrence while undergoing active management of the other cancer (Table 3). None of these patients had a known germline mutation. In all cases, the sequence of diagnosis, the stage of each cancer at diagnosis, and the overall prognosis of each cancer impacted clinical decision-making. Figure 1 summarizes key takeaways and general management workflow for these difficult cases. In general, the cancer that was most life limiting was prioritized while maximizing concurrent treatment. For example, for patient 3, FOLFOXIRI was given for metastatic CRC in addition to trastuzumab for stage 1 HER2+ breast cancer. A common therapeutic dilemma encountered was whether to continue hormonal therapy during fluorouracil (5-FU) containing chemotherapy for CRC, with observation of a range of practice variations. Multiple patients discontinued hormonal therapy during FOLFOX or FOLFIRI treatment since 5-FU is also active against breast cancer. Although neuropathy is a dose-limiting side effect of both docetaxel and cyclophosphamide for breast cancer and oxaliplatin for CRC, 2 patients tolerated full treatment with both regimens.

**Table 3.** Management nuances of patients with both breast cancer and CRC.

Number	Malignancy 1 (age at diagnosis)	Malignancy 2 (age at diagnosis)	Therapeutic dilemma	Management strategy	Outcome^a^
1	*Breast cancer (47)* 2009: dx with stage 2a ER+/PR+/HER2− IDC, underwent mastectomy with adjuvant TC + anastrazole 2014: recurrence to LNs started on exemestane 2016: (after CRC diagnosis) recurrence to supraclavicular LN started radiation and fulvestrant 2021: recurrence in axillary LN s/p excision and put on letrozole	*Colorectal cancer (53)* 2016: dx with stage 3 transverse colon cancer treated with resection and adjuvant CapeOx	What to do with hormonal therapy for breast cancer while receiving CapeOx for colon cancer?	Held fulvestrant while on CapeOx since capecitabine is an effective tx for metastatic breast cancer	Alive without evidence of breast cancer after 2021 recurrence and without colon cancer recurrence after 5 years of surveillance
2	*Breast cancer (41)* 2007: dx with stage 2 ER+/PR+/HER2− IDC tx with mastectomy followed by adjuvant TC, followed by goserelin/tamoxifen, then maintenance tamoxifen for 5 years, followed by letrozole	*Colorectal cancer (46)* 2013: dx with stage 4 (BRAF mutant) transverse colon cancer with metastases to the liver treated with colectomy, hepatectomy, and FOLFOX + bevacizumab	What to do with maintenance hormonal therapy while receiving FOLFOX? How does prior chemotherapy for breast cancer treatment impact choice of chemotherapy for CRC?	Continued hormonal therapy while receiving FOLFOX Considered the side effects from prior breast cancer tx: although both taxotere and oxaliplatin cause neuropathy, oxaliplatin was tolerated for all 13 cycles	Alive without breast recurrence, and 8 years without evidence of recurrent or metastatic colon cancer
3	*Colorectal cancer (35)* December 2018: dx with stage 4 ascending colon cancer with liver metastases (ERBB2 negative, RAS mutated; staging scans reveal synchronous breast cancer) Treated with FOLFOXIRI (+trastuzumab), then hepatectomy (with concurrent mastectomy), followed by FOLFOXIRI (+trastuzumab and tamoxifen) October 2019: recurrence to lung and liver with subsequent progression on all further lines of therapy	*Breast cancer (35)* January 2019: dx with stage 1a ER+/PR+/HER2+ IDC, treated with trastuzumab initially then mastectomy while undergoing surgery for colon cancer, then continued trastuzumab and tamoxifen October 2019: tamoxifen and trastuzumab stopped due to the aggressive nature of colon cancer	With synchronous breast and colon cancer, which cancer should be treated first or how to treat both at the same time? When to discontinue hormonal and trastuzumab therapy for early-stage breast cancer given alongside chemotherapy for mCRC?	Since the colon cancer was more advanced it was treated first Since the breast cancer was HER2+ and hormone receptor positive, HER2 and hormonal therapy were added to the colon cancer chemotherapy regimen When surgery was done for the colon cancer, concurrent mastectomy was performed Since the breast cancer had low risk for recurrence, hormonal therapy and trastuzumab were stopped when colon cancer was progressing and life limiting	Deceased at age 38 from metastatic colon cancer
4	*Colorectal cancer (39)* July 2019: dx with rectal cancer (KRAS mutated) metastatic to the liver, LN including L axillary, vaginal cuff, peritoneum, and with suspicious breast uptake, tx with neoadjuvant FOLFOX followed by LAR with TAH/BSO; peritoneal implants found January 2020: started adjuvant FOLFOX + bevacizumab (+hormonal therapy) with progression November 2020: started FOLFIRI + bevacizumab February 2021: progression of both CRC and breast cancer; restarted on FOLFIRI + bevacizumab with response (then held for breast surgery), with subsequent rapid progression off therapy so restarted FOLFIRI + bevacizumab (+exemestane) followed by progression on all further therapies	*Breast cancer (39)* August 2019: breast biopsy for suspicious uptake on PET/CT for CRC: dx with ER+/PR+/HER2− IDC with axillary LN involvement November 2019: started tamoxifen after neoadjuvant FOLFOX and then switched to anastrazole after TAH/BSO February 2021: progression of breast cancer, anastrazole stopped, surgery deferred while receiving FOLFIRI for CRC October 2021: lumpectomy with residual disease and matted nodes, deferred XRT and started exemestane	When there is a progression of both metastatic rectal cancer and locally advanced breast cancer what to treat first? Should anastrazole be continued when starting FOLFIRI? What to do about postoperative radiation therapy for residual breast cancer while undergoing tx for mCRC?	Since metastatic rectal cancer had a worse prognosis, chemotherapy was started first and only after achieving rectal cancer control was chemotherapy paused and surgery intended to cure the breast cancer undertaken to increase her candidacy for CRC clinical trials Given her breast cancer progressed while on anastrazole it was stopped during FOLFIRI given 5-FU has activity against breast cancer Even though her breast cancer qualified for radiation treatment since she had residual disease, radiation was deferred given the urgency to restart mCRC tx	Deceased at age 42 from metastatic rectal cancer
5	*Colorectal cancer (53)* June 2009: dx with stage 1 sigmoid colon cancer detected on routine colonoscopy; treated with sigmoidectomy	*Breast cancer (53)* September 2009: dx with stage 1 ER+/PR−/HER2− IDC treated with lumpectomy, adjuvant TC and radiation therapy	Does surgery for colon cancer (stage 1) <2 months prior change the tx for a new diagnosis of breast cancer?	The tx of her stage 1 breast cancer was not impacted by her colon cancer since it was stage 1 (resected) and no adjuvant treatment was indicated for CRC	Alive with no evidence of colon or breast cancer recurrence 10 years after diagnosis
6	*Colorectal cancer (38)* 2018: dx with stage 3 rectosigmoid cancer s/p resection and adjuvant FOLFOX 2020: developed pulmonary metastatic disease and lymphadenopathy; started on FOLFIRI + bevacizumab	*Breast tumor (40)* 2020: new breast nodule identified at the time of mCRC recurrence 2020: elected to get a biopsy which showed a fibroepithelial lesion 6 months later, a mammogram showed growth of the lesion	What to do with a new growing breast lesion while undergoing tx for mCRC? How to address pulmonary metastasis with 2 possible cancers as the primary?	Despite growth, the decision was made not to resect the breast lesion since that would have required holding CRC tx, which was a more life-limiting diagnosis Biopsy of pulmonary metastases was considered but ultimately not recommended given known mCRC and most fibroepithelial lesions are benign	Deceased at age 43 from metastatic rectal cancer
7	*Breast cancer (48)* 2018: (perimenopausal) dx with high-grade DCIS s/p breast lumpectomy and adjuvant radiation and started on tamoxifen	*Colorectal cancer (51)* 2021: dx with stage 3B colon cancer abutting the appendiceal orifice; underwent surgical resection and started on adjuvant FOLFOX × 6 months	What to do with tamoxifen therapy for high-grade DCIS while on adjuvant FOLFOX for CRC?	Tamoxifen for DCIS was held during FOLFOX to reduce risk of thrombotic complications	Alive without breast cancer recurrence and no evidence of colon cancer; in surveillance

^a^Data cutoff was December 19, 2022.

Abbreviations: dx, diagnosed; tx, treatment; ER, estrogen receptor; PR, progesterone receptor; IDC, invasive ductal carcinoma; TC, taxotere Cytoxan; LN, lymph; node; CapeOx, capecitabine oxaliplatin; FOLFOX, leucovorin calcium (folinic acid), fluorouracil, oxaliplatin; LAR, low anterior resection; TAH/BSO, total abdominal hysterectomy, bilateral salpingoophorectomy; mCRC, metastatic colorectal cancer; s/p, status post; FOLFIRI, leucovorin calcium (folinic acid), fluorouracil, and irinotecan hydrochloride; XRT, radiation therapy; DCIS, ductal carcinoma in situ.

## Discussion

This is the first study to characterize the demographic, clinical, and genetic features of younger patients diagnosed with both breast cancer and CRC. In addition, this study describes therapeutic dilemmas and management nuances that could help guide future physicians in the treatment of this complex group of patients.

We found that the age and stage of younger patients with both breast cancer and CRC followed the trends from previous reports of young-onset patients with either cancer.^2-5^ Specifically, the median age of diagnosis of breast cancer was 43 and colon cancer was 50 in our study, compared to reported median ages of 63 (28% diagnosed before age 55) and 66 (13% diagnosed before age 55), respectively.^2-5^ As expected, breast cancer was more commonly diagnosed before CRC in this cohort. Additionally, a SEER study of patients of all ages with both breast cancer and CRC also showed breast cancer was more often diagnosed first.^10^ Most patients in this study had early-stage breast and later-stage CRC. Young patients diagnosed with breast cancer mostly have local or regional disease, while early-onset colorectal cancer is characterized by advanced stage at diagnosis.^2,21^

We conducted a comprehensive review of medical records in an effort to identify risk factors within this cohort and found alcohol consumption and sedentary jobs were the most common. The majority of patients in this study had a risk factor for at least one of the cancers. Almost 50% of patients in this study were current alcohol consumers, although the amount of alcohol was not recorded. Studies have shown that even modest amounts of alcohol can increase the risk of both breast and CRC and therefore could contribute to increased risk in this population.^7,22-25^ A substantial group of patients in this study (34%) had sedentary jobs which may also confer an increased risk of CRC and breast cancer.^26-30^ Research shows that, in the United States, less than 20% of occupations require moderate-intensity physical activity, therefore the majority of employed individuals have sedentary jobs and this is not unique to our study population.^31^ There are many potential confounders, including the association between sedentary jobs and health care access, which make the correlation between sedentary jobs and cancer risk difficult to interpret. Only a small subset of patients in this study were obese. While obesity has been shown to almost double the risk of early-onset CRC, higher BMI has been associated with lower breast cancer incidence in premenopausal women and increased risk in postmenopausal women.^25,32-36^ Although inflammatory bowel disease is a clear risk factor for CRC, a large meta-analysis showed that IBD had no influence on breast cancer risk.^37-39^ Further studies are needed to understand what role these risk factors are playing in the development of both cancers in this young population.

Due to the retrospective nature of this study, there were variable rates and types of genetic testing done for patients in this study. Acknowledging this limitation, 24% of patients in this study had germline mutations in either BRCA (12%) or Lynch syndrome (12%). In the literature, BRCA1 and BRCA2 are found in approximately 10%-20% of patients with early-onset breast cancer and approximately 13% of patients with early-onset CRC have a hereditary cancer (most commonly Lynch syndrome: 8%).^40,41^ Additionally, although not seen in the current study, germline mutation of CHEK2 has been associated with both breast and CRC.^20,42^ In clinical practice, early-onset of either disease should lead to genetic testing. In this study, 37% of patients had a first-degree relative with either breast (22%) or CRC (15%). This is compared to reports of 13% of people with breast cancer have a family history of breast cancer in a first-degree relative and approximately 7%-26% of early-onset CRC have a first-degree relative with CRC.^43-46^ Understanding the family history patterns of young patients with both cancers could help inform screening for both cancers in the future.

This study provides key insights about some of the management dilemmas that physicians face when treating a patient with both breast cancer and CRC. In general, the treatment strategy is highly individualized and depends on several factors including (1) order of diagnosis, (2) stage of each cancer, and (3) overall prognosis. In general, the cancer that was more life limiting was often treated first, but when possible, concurrent treatments were given. This study had more early-stage breast cancer and later-stage CRC. Therefore, although the survival outcomes with metastatic breast cancer and mCRC are similar, ie, median overall survival of ~3 years and 5-year survival ~30%, the late-stage CRC was often the priority cancer to treat in this cohort.^47-49^ Occasionally, the early-stage cancer was treated with curative intent to increase the eligibility for clinical trials, since clinical trials typically exclude patients with a concurrent active cancer; however, 41% of trials exclude prior cancer within 5 years.^50^ When possible, combined surgeries were planned to address both the breast and CRC.

The fluoropyrimidine class of chemotherapy has shown efficacy in both breast and CRC, and thus was a frequently selected treatment in this study with the goal to treat both cancers.^51^ Attention was given to overlapping toxicities of CRC and breast cancer treatment, particularly neuropathy from both platinum chemotherapies and taxanes.^52-54^ However, the patients in this study were able to complete all cycles of oxaliplatin after prior taxane therapy without dose-limiting toxicities. Patients should be monitored for overlapping toxicities if treating a second primary cancer with additional chemotherapy.

A common dilemma encountered was whether to continue hormonal therapy for breast cancer maintenance therapy during chemotherapy treatment for CRC. According to the Adjuvant Tamoxifen: Longer Against Shorter (ATLAS) Collaborative Group, patients with early hormone-positive breast cancer should continue hormonal therapy for up to 10 years.^55^ Therefore, most younger patients with either synchronous or metachronous CRC and breast cancer face this dilemma. In this study some oncologists held hormonal therapy during CRC-directed chemotherapy, due to the fact that the fluoropyrimidines (especially 5-fluorouracil and capecitabine) have been shown to have activity against breast cancers, while others continued hormonal therapy even during these treatments.^51^ In general, when treating patients with breast cancer with cytotoxic chemotherapy, hormonal therapy is withheld due to the theoretical risk that endocrine therapy which is cytostatic and prevents cells from dividing could decrease the efficacy of chemotherapy which targets actively dividing cells, although this has not been proven.^56^ Since the chemotherapy agents are targeting CRC and the endocrine therapy is targeting the breast cancer, it is unclear whether the same principal applies. One oncologist held tamoxifen during FOLFOX therapy due to the theoretical increased risk of venous thromboembolism (VTE) from chemotherapy and tamoxifen therapy together. While there are no guidelines in this space, one study showed a higher risk of VTE in patients with breast cancer after tamoxifen and chemotherapy, however, not after aromatase inhibitor therapies.^57^

Another consideration for patients with synchronous mCRC and breast cancer is to determine the HER2 (ERBB2) status of the mCRC, as approximately 3% of mCRC are HER2 positive, and 5% of KRAS and NRAS wild-type tumors.^58^ Similar to breast cancer, HER2 amplified mCRC can indicate more aggressive disease. Although studies of HER2-targeted therapy for mCRC have encouraging signal, they are limited due to the low rate of ERBB2 overexpression. However, if the patient has HER2 positive breast cancer, the utilization of ERBB targeted therapy may offer therapeutic benefit for both cancers.^59^ This study was conducted prior to the approval of tucatinib for mCRC and therefore was not used for patients in this study.^60^ The optimal choice of HER2 targeted therapy with a dual diagnosis of breast and CRC has not yet been determined.

This study has several limitations that must be acknowledged. Particularly given the modest sample size and retrospective design this study is purely exploratory in nature, although it is one of the biggest sample sizes of this rare population. Since some of the patients were identified through the UCSF Cancer Genetics and Prevention Program Registry, selection bias could contribute to the high number of germline mutations among this population. Additionally, the number of genes tested over the study period was variable, so this study could underestimate the true number of hereditary cancers among this population. No conclusions about the causality of the risk factors or germline mutations could be made since there was no control group for comparison. The medical records had a high degree of missing data, particularly for hormone receptor status of breast cancers, a limitation of retrospective data collection. We also lacked detailed information about the amount of alcohol or tobacco use. Additionally, there have been significant changes to the standard of care over the course of this study to the treatment of each cancer, which could change comanagement decisions of these patients in the future.

## Conclusion

Younger patients with both breast cancer and CRC are a unique cohort, many without known risk factors. Alcohol consumption and sedentary jobs were the most common risk factors identified among patients without a known genetic predisposition. Comanagement of both cancers requires individualized, multidisciplinary care. As the incidence of both cancers is increasing in adults under the age of 50, future studies are needed to fully characterize this growing group of patients.

## Data Availability

The data underlying this article will be shared on reasonable request to the corresponding author.
